# Supplementary material for: Are carbon emissions trading and green financial instruments synergistic? -Comprehensive quantitative research based on content analysis
Source: PLoS One. 2024 Mar 7;19(3):e0298601. doi: 10.1371/journal.pone.0298601 (PMC10919601; doi:10.1371/journal.pone.0298601)
Supplement: S1 Data — (ZIP) [file pone.0298601.s001.zip › Data/地级市Z_F工作报告.zip!/╣π╢1⁄2/╒┐╜¡╩╨/└┤╫╘╣1⁄2╓┌║┼í╛┬φ┐╦╩2╛▌═°í┐_2007.docx]

各位代表：

　　我代表市人民政府向大会作政府工作报告，请予审议，并请市政协委员和其他列席的同志提出意见。

　　2006年及本届政府任期工作回顾

　　2006年是全面实施“十一五”发展规划的第一年。一年来，市政府在中共湛江市委的正确领导下，在市人大及其常委会和市政协的监督支持下，坚持科学发展、以人为本的执政理念，着力推动经济社会发展，经济建设提速增效，社会事业全面进步，人民生活持续改善，圆满完成了市十一届人大四次会议确定的各项任务，实现了“十一五”的良好开局。

　　经济总量迈上新台阶。预计全市完成生产总值770亿元，净增112亿元，增长12.8%，增速创11年来新高。其中，第一产业增加值165亿元，增长7.5%；第二产业增加值355亿元，增长15.2%；第三产业增加值250亿元，增长13.2%。人均生产总值11500元，增长10.3%。单位生产总值能耗下降3%。三次产业比例调整为21.4∶46.1∶32.5。地方财政一般预算收入30.4亿元，增长26.6%；来源于湛江的财政总收入146.8亿元，增长24.6%。

　　工业发展增添新后劲。预计全市完成工业总产值950亿元，增长15.5%；工业增加值325亿元，增长14.9%。其中，规模以上工业总产值814亿元，增长17.2%；增加值266亿元，增长15.1%。单位工业增加值能耗下降2.8%。以重化工业为龙头的临港工业迅速壮大，实现产值418亿元，增长23%。工业企业经济效益综合指数285.6%，位列全省第一。工业对生产总值增长的贡献率47.6%，提高3个百分点。奥里油电厂、湛江珠啤20万吨易地改造、环球钢轮首期等67个工业项目建成投产或试产。新增省级以上高新技术企业15家，高新技术产品实现总产值135亿元，增长24%。民营工业增加值117亿元，增长28.8%。35个工业产品获得国家、省各类名牌称号。我市被命名为“中国电饭锅产业基地”。经济技术开发区水产加工区成为国家级水产加工示范基地。湛江经济技术开发区扩区和广东湛江临港工业园区获得国家批准。

　　新农村建设取得新成效。全市各方面投入新农村建设资金48.7亿元，增长45%。建成市级生态文明村330个和生态文明示范村20个；我市社会主义新农村建设经验在全国、全省推广。全市农业总产值267亿元，增长7.6%。粮食总产量167.6万吨，完成了省下达的考核任务。甘蔗产量1000万吨，蔗农增收10.7亿元。水果、蔬菜和水产品产量居全省前列，对虾加工出口量居全国首位。畜牧业快速发展，成为全省最大的活牛出口基地。国家决定建立广东省湛江海峡两岸农业合作试验区，并全面启动建设。新增市级农业龙头企业13家，农业龙头企业销售收入107亿元，带动37.3万农户，户均增收2900元。农民专业合作经济组织发展到141个。40个农海产品获得国家、省名牌称号或质量认证。设立10万亩国家级基本农田保护示范区。启动实施22宗城乡水利减灾防灾工程，基本完成徐闻大水桥水库和49宗小型水库除险加固工程。完成村道路面硬化改造2387公里。绿化造林24.2万亩，中荷合作雷州半岛红树林项目圆满竣工。

　　县域经济迈出新步伐。县域完成生产总值330亿元，增长12.3％。各县(市)规模以上工业增加值、固定资产投资、社会消费品零售总额等主要经济指标实现两位数增长。县域财政一般预算收入增幅达27.5%，可望全部获得省财政激励机制奖。县域民营经济发展迅速，实现增加值172亿元，增长13.9％，占县域生产总值52％。

　　基础设施和城市规划建设取得新进展。全社会固定资产投资完成205亿元，增长13.6％，投资总量创历史新高。全年在建重点项目26个，完成投资51.9亿元，完成年度计划103.8％。海湾大桥、疏港公路一期工程建成通车，黎湛铁路河唇至湛江段增建二线加快建设。500千伏港城输变电工程竣工投产。完成国道207线廉江良垌至遂溪一级公路、麻斜国防战备公路、东海岛进港公路改造，建成县通镇公路120.6公里、镇通村委会公路路面硬化1800公里，全市公路通车总里程8487公里。完成城镇体系规划、城市近期建设规划和19个中心镇总体规划修编，编制湛江经济技术开发区、东海岛分区和坡头海东新区等控制性详细规划。推进土地储备，全年入库土地1760亩，清理闲置土地91宗。积极创建国家卫生城市和国家环保模范城市。湖光岩获得世界地质公园称号。完成金康路、金川路、乐山大道等6条主要道路和一批小街小巷改造；加快实施百蓬路、南方路网首期、滨湖路一期、体育南路东段、体育北路东段和椹川东二路等新建或改造工程；有序推进市区旧城及城中村改造。完成金沙湾观海长廊二期、绿塘河公园二期、金康榕园建设和霞湖公园改造等20多项绿化工程，建成13个小绿地、小游园，南国热带花园、中澳友谊花园和绿塘河公园三期等工程进展顺利，市区新增绿地103万平方米。完成赤坎江一期整治、霞湖排水截污、市生活垃圾场一期封场等工程，文保河、绿塘河排水截污工程顺利推进，湛江电厂首台机组脱硫装置投入运行，主要污染物化学需氧量和二氧化硫排放量分别降低1%和1.2%。城市环境质量继续排在全国环保重点城市前列。建成城市管理投诉热线服务系统，城市管理有效改善。

　　第三产业发展水平有新提升。港口物流、交通运输、商贸流通、金融保险、旅游、房地产等重点服务行业快速发展，消费需求不断扩大。全市港口货物吞吐量8160万吨，增长23.3%，创历史新高；其中湛江港集团公司货物吞吐量突破5000万吨，增长22%。公路、铁路货运量完成4300万吨和2129万吨,分别增长9%和10.9%。消费市场购销两旺，预计全市社会消费品零售总额317亿元，增长18%，创11年来新高。预计全年接待游客680万人次，增长19.3%；旅游业总收入37亿元，增长15%。成功举办2006湛江工业博览会，签订投资合同170亿元。

　　外向型经济有新发展。预计全市外贸出口12亿美元，增长25.7%，创历史新高；3个县（市、区）外贸出口超1亿美元。吸收外资稳步发展，一批重点外商投资项目建成投产或加快建设，全市实际吸收外资5500万美元，增长41.1%。

　　体制改革有新推进。初步建立以保值增值为目标的国有资产监管体系，28家劣势企业退出市场。基本完成行政审批事项清理审查工作。实行投资核准制和备案制。调整市对区财政体制，拓展非税收入征管范围。进一步理顺垃圾处理、自来水、天然气、有线电视、市区交通价格。进一步完善农户土地经营权流转机制。市粮食、供销系统及金融改革进展顺利，农村信用社超额完成增资扩股任务，城市信用社债务置换和退市工作取得新进展，市商业银行成功重组。

　　社会事业有新进步。科技：实施市级科技计划中标项目42个、粤港关键领域重点突破中标项目5个；新建两家市级工程中心、3家市级企业技术开发中心，15家工业企业建立技术开发机构；与中山大学建立联合实验室、检测中心和5个产学研合作基地；取得各类科技成果65项、专利申请520项和授权325项。教育：实施农村义务教育免收学生杂费，直接减轻农民负担3.8亿元；改造148所农村义务教育学校生活设施和13所市区学校；湛江一中培才学校、市二中新校区建成使用；4所重点中学通过国家级示范性高中初期评估验收；职业教育与成人教育快速发展；高考本科上线人数和高分优秀生人数再创新高。文化：雷州石狗被列入国家民俗民间文化保护工程，遂溪醒狮和东海人龙舞被列入国家首批非物质文化遗产名录，6个民间文化艺术项目入选省级非物质文化遗产代表作名录，完成50户以上的自然村“村村通广播电视”建设任务。卫生：全市公有医疗单位设立平价医院4所、平价门诊162间，增设社区卫生服务机构9个，基本完成37间乡镇薄弱卫生院改造；农村人口参加新型合作医疗覆盖率61.7%；有效预防和控制禽流感、艾滋病等重大传染病。体育：湛江籍运动员参加第十五届亚运会等国际大赛获得23枚金牌；在省第十二届运动会上获得59.5枚金牌，总分名列全省第八位，获省体育突出贡献奖。圆满完成省下达的人口与计划生育目标管理责任制各项任务，全年人口自然增长率6.68‰。完成市区人防规划编制并实现县（市）与市区防空同步警报。人才引进和人才资源开发取得新成效。双拥共建与优抚安置、国防动员、兵员征集和民兵预备役工作不断加强，积极为驻湛部队解决实际问题，军政军民更加团结。完成全国1%人口抽样调查任务。电子文档建设加快推进。工商、质监、食品药品监督等部门打假治劣、整顿和规范市场经济秩序以及保障食品安全工作卓有成效，食品安全监管体系进一步完善。外事、侨务、对台等部门积极加强与港澳台、海外华侨社团和国外的经济文化交往。海关、检验检疫、海事、边检、铁路、民航等中央和省驻湛单位为全市经济发展作出了新贡献。气象、地震、民族宗教、残联、扶贫和地方志等工作取得新成绩。

　　人民生活水平有新提高。预计城镇居民人均可支配收入10450元，增长8.3%；农民人均纯收入4620元，增长8.7%。城镇、农村居民人均住房面积分别为28平方米和26平方米，均达全国平均水平。全市参加社会保险56.1万人，增加6.5万人，增长6%。企业养老保险基金首次实现收支平衡。新增就业岗位5.5万个，城镇登记失业率3.2%。农村劳动力转移就业25.3万人。多渠道筹措资金，解决15.2万城乡群众最低生活保障和5.5万“五保户”供养问题。改造乡镇敬老院16间，新建村级敬老院136间。完成省农村安居工程7400户住房建设和城镇特困户住房解困任务，改造水库移民危房3127户，全市建成廉租房2.5万平方米，住房公积金发放个人贷款1.4亿元，城乡部分住房困难群众居住条件得到改善。居民储蓄存款余额567亿元，比年初增长12％。

　　民主法制和精神文明建设取得新成果。办复省市人大代表议案及建议148件、政协委员提案139件，满意率98%。深入实施行政许可法，进一步撤减行政许可事项，办理行政复议案件116宗，有效维护了公共利益和社会秩序。政务公开制度全面推进，廉政建设不断加强，认真履行市政府向社会公开作出的廉政承诺。实行审计结果公告制度，强化监察工作，切实纠正损害群众利益的不正之风。普法教育、法律援助、劳动教养和刑释解教人员安置帮教工作有新发展。加强社会治安综合治理，依法严厉打击各种严重违法犯罪和黑恶势力，社会治安保持稳定。深入开展反走私综合治理，有效遏制走私活动。认真处理群众来信来访，人民调解组织网络不断完善，有效化解各种社会矛盾。安全生产形势稳定，全面完成省下达的控制考核指标任务。广泛开展群众性精神文明创建活动，评选表彰市文明行业3个、文明单位标兵20个、文明单位91个、文明社区6个、生态文明镇12个。

　　各位代表！

　　本届政府任期四年来，坚持以邓小平理论和“三个代表”重要思想为指导，坚持以科学发展观为统领，认真贯彻党的十六大和十六大以来历次中央全会精神，认真贯彻胡锦涛总书记视察湛江的重要指示精神，认真贯彻张德江书记、黄华华省长关于湛江加快发展的重要指示和省委、省政府的工作部署，坚决执行市委的各项决策，围绕建设现代化新兴港口工业城市和美丽的南方海滨城市的目标，全力实施“工业立市、以港兴市”发展战略，紧紧依靠全市人民，恪尽职守，团结实干，迎难而上，圆满完成了市第十一届人民代表大会各次会议提出的目标任务，圆满完成了“十五”计划的各项预期指标，实现了国民经济从农业支配型到工业主导型的历史性跨越、人民生活从温饱到小康的历史性跨越，开启了我市科学发展的新时期。2006年与2002年相比，全市生产总值净增345亿元，增长54.7%；人均生产总值净增4581元，增长47%；三次产业结构不断优化，第一产业比重下降5.9个百分点，第二产业比重提高10.7个百分点，其中工业增加值比重提高11.1个百分点；地方财政一般预算收入净增14.9亿元，增长96.3%；来源于湛江的财政总收入净增84.2亿元，增长134.4%；全社会固定资产投资净增110亿元，增长114.6%；港口货物吞吐量净增4574万吨，增长127.6%；社会消费品零售总额净增142亿元，增长80.9%；外贸出口净增6.5亿美元，增长119.8%；实际吸收外资不断扩大，先后有15家世界500强企业和跨国公司在湛投资兴业；人民生活水平持续提高，城镇居民人均可支配收入净增2806元，农民人均纯收入净增1068元，分别增长36.7%和30.1%。糖业、公交、公共财政、行政体制、地方金融等领域改革取得显著成效。城乡建设取得重大成就，完成了30万吨级油码头、25万吨级深水航道、20万吨级铁矿石码头、广湛和渝湛高速公路以及椹川路、机场路、东新路改造等一批重大基础设施建设项目，建成了渔港公园、金沙湾观海长廊、海田公园、南桥和北桥公园等大型绿化项目以及一批小游园、小绿地，累计新增城市公园绿地279万平方米；城市环境质量连续4年排在全国环保重点城市前三位，先后荣获中国优秀旅游城市、国家园林城市、广东省卫生城市、广东省文明城市等称号；以创建生态文明村为先导的社会主义新农村建设取得丰硕成果，广大农村面貌发生深刻变化，全市建成各级生态文明村3740个，占自然村总数的28.3%。科技、教育、文化、卫生、体育等各项社会事业全面进步。

　　过去四年湛江的发展，使我们从理论和实践上，进一步提高了对实践“三个代表”重要思想、落实科学发展观、构建和谐社会的认识，进一步深化了对我市现代化建设特点和规律的认识，得到了许多对今后发展具有指导意义的经验和启示。第一，必须把科学发展作为第一要务。始终坚持以经济建设为中心不放松、不动摇、不分神，集中精力发展生产力，在科学发展观指引下，转变发展观念，创新发展模式，提高发展质量，努力实现科学发展、协调发展、和谐发展。第二，必须把“工业立市、以港兴市”作为核心发展战略。始终坚持以工业化为核心，加快工业化进程，通过工业化带动第三产业，提升第一产业，促进经济转型结构优化，打造跨越发展的坚强支柱；始终坚持充分发挥港口优势，加快南方大港建设，增强港口的龙头带动作用，打造跨越发展的强大引擎。第三，必须把重点项目建设作为加快发展的重要支撑。始终把抓项目就是抓机遇、抓发展的理念贯穿于经济社会发展的各项工作之中，通过全力推进重点项目建设，保持足够的投资强度，形成对经济增长的强劲拉动；不断完善基础设施和城市功能，提升发展优势，增强发展后劲。第四，必须把统筹协调作为实现科学发展的重要途径。始终紧紧抓住“五个统筹”这一科学发展观的根本要求，着力加强县域经济、“三农”工作、社会事业、生态建设等薄弱环节，增强发展的协调性，促进经济增长与人口、资源、环境相适应，发展速度与结构、质量、效益相统一，努力实现可持续发展。第五，必须把改革开放和自主创新作为加快发展的内在动力。始终坚定不移地推进改革，不断消除发展障碍；坚定不移地扩大开放，不断增强发展活力；坚定不移地推进科技进步和自主创新，为经济社会发展提供强大的智力支持。第六，必须把构建和谐社会作为发展的根本目标。始终坚持发展为了人民、发展依靠人民、发展成果由人民共享，着力解决广大群众最关心、最直接、最现实的利益问题，积极化解历史遗留问题和突出矛盾，努力促进社会公平正义，维护社会稳定，致力建设老百姓城市和社会主义新农村，让发展成果更多地惠及最广大人民群众。

　　过去四年本届政府取得的成就，是中共湛江市委正确领导的结果，是各级人民代表大会及其常委会和人民政协监督支持的结果，是中央、省驻湛单位、驻湛部队和社会各界共同努力的结果，是全市人民艰苦奋斗的结果。在此，我代表市人民政府，向各级人大代表和政协委员，向在各个岗位上辛勤劳动、作出贡献的广大工人、农民、知识分子、干部、驻湛人民解放军、武警部队官兵、人民警察和社会各界人士，向所有关心和支持湛江建设发展的港澳台同胞、海外侨胞和国际友人，表示崇高的敬意和衷心的感谢！

　　在充分肯定成绩、增强信心的同时，我们也清醒地看到，我市经济社会发展中还存在不少困难和问题：一是经济发展总体水平仍然不高，经济总量不够大，人均生产总值偏低，财政实力相对薄弱。二是产业结构和比重仍然不够合理。工业支柱产业链条不长，带动力不强，高新技术产业比重偏低，第三产业发展不充分，尤其是现代服务业发展较慢。三是城乡、区域发展还不够协调，县域经济总量偏小，工业规模不大，缺乏大项目支撑，发展后劲不足；乡镇经济发展滞后，财力薄弱。四是城市管理水平有待提高，综合运用经济、法律、行政手段统筹解决城市管理实际问题的力度不够，局部环境脏乱差和交通不够畅顺问题突出，治理任务艰巨。五是社会建设还存在薄弱环节。教育、卫生、群众文化等社会事业还不适应人民群众日益增长的需求。就业压力仍然较大，社会保障能力较弱，部分低收入群众生活还比较困难。六是政府职能和机关作风的转变尚未适应经济社会快速发展的要求，一些政府工作人员服务意识淡薄，工作责任感不强，办事效率不高，积极进取、主动负责的精神不足。我们要高度重视存在的问题和困难，进一步增强忧患意识，采取切实有效措施加以解决，努力把各项工作做得更好。

　　今后五年奋斗目标和2007年主要任务

　　今后五年是湛江实现跨越发展、全面建设小康社会的关键时期。我们站在新的历史起点上，面向未来，肩负承前启后、继往开来的重大历史责任，必须抓住机遇，乘势而上，努力推动改革开放和现代化建设事业再上新台阶，再创新辉煌。

　　今后五年的奋斗目标是：全面完成“十一五”规划预期目标，实现“十二五”良好起步。全市生产总值年均增长11%以上，2011年达到1350亿元，人均生产总值达19000元（约2500美元）。工业总产值年均增长14%，达1700亿元以上。固定资产投资年均增长12.5%，累计1400亿元。外贸进出口总值年均增长13%，达36亿美元，其中出口年均增长12%，达19亿美元；实际吸收外资年均增长15%，达1亿美元。地方财政一般预算收入年均增长15%，达57亿元；来源于湛江的财政总收入年均增长13%，达250亿元。构建区域性国际航运中心、物流中心，全市港口货物吞吐量年均增长13%，2008年突破1亿吨大关，2011年达1.5亿吨。单位生产总值能耗年均降低3%左右。信息化综合指数60%以上。加快建设社会主义新农村，具有湛江特色的生态文明村达到自然村总数的50%以上，农民人均纯收入年均增长6%。城镇化水平提高到50%以上，城镇居民人均可支配收入年均增长8%，城镇登记失业率控制在3.8%以内。人口自然增长率控制在7‰以内。市区年均增加公共绿地面积100万平方米，人均公共绿地面积达12平方米以上，城市环境质量保持在全国环保重点城市前五名。争创国家卫生城市、国家环保模范城市、国家生态园林城市、全国绿化模范城市和全国文明城市，继续保持全国双拥模范城称号。

　　今年市政府工作的总体要求是：坚持以邓小平理论和“三个代表”重要思想为指导，全面落实科学发展观，认真贯彻市第九次党代会精神，以建设现代化新兴港口工业城市和美丽的南方海滨城市为目标，深入实施“工业立市、以港兴市”发展战略，优化经济结构，转变增长方式，深化改革开放，推进自主创新，壮大县域经济，加强社会建设，改善人民生活，维护社会稳定，促进社会和谐，着力推动经济社会转入科学发展轨道，为实现湛江跨越发展而努力奋斗

　　今年我市经济和社会发展主要预期目标是：全市生产总值增长11%，其中第一产业增长5%，第二产业增长12.5%（工业增长12.5%），第三产业增长13%；单位生产总值能耗降低3%左右；主要污染物排放总量减少5%；全社会固定资产投资增长10%；外贸出口和实际吸收外资分别增长15%；地方财政一般预算收入增长12%；居民消费价格涨幅稳定在2.5%以内；城镇登记失业率控制在3.5%以内；人口自然增长率控制在7‰以下；城镇居民人均可支配收入增长8%，农村居民人均纯收入增长6%。

　　今年的主要任务和工作重点是：

　　一、坚持“工业立市”不动摇，推进产业结构优化升级

　　推进重点项目建设增强工业发展后劲。全市安排重点建设项目33个，年度计划投资50亿元。完善政府引导、市场运作、主体到位的项目建设机制，促进项目建设程序化、规范化和科学化。严格落实各级领导联系重点项目和部门责任制度，将任务细化分解落实到具体领导、具体单位和责任人，排出建设时间表，做到领导抓督促指导，部门抓综合服务，业主抓具体建设，形成推进重点项目建设的强大合力。扎实推进湛江钢铁等重大项目前期工作。认真跟踪落实国家和省规划在湛投资建设的重点项目。

　　推进园区建设培育新的工业增长点。高起点、高标准、高水平做好湛江经济技术开发区东海岛新区和广东湛江临港工业园区规划，按照统一规划、基础先行、重点带动、节约高效的原则，实现滚动发展，当前要集中力量做好征地工作，全力推进基础设施建设。湛江经济技术开发区要充分发挥东海岛新区优势，抓住一批上规模、有影响的临港工业项目，迅速打开局面。各县（市、区）开发区建设要坚持与经济结构调整、培育新型产业、发展区域特色经济和促进城镇化相结合，推进园区土地集约化经营、产业链条式延伸、项目集群式组合、资源循环式利用，加大力度招商引资，加快项目建设速度，进一步提高开发质量和效益，增强聚集和带动力。全市园区工业产值占全市工业总产值的比重提高到45%。

　　推进企业集群化壮大工业支柱产业。坚持把培育产业集群作为推进工业化的重要举措，围绕“两大重点”和“八大支柱”，推进一批具有地方特色的优势产业集群建设。一是大力发展临港重化产业。重点支持中海油湛江分公司、中石化东兴炼油、中石油燃料油储备库、奥里油电厂等大型企业加快发展，推动建设一批石化、电力等组团式项目以及开发上下游产品，拉长壮大产业链，打造临港重化工业核心区。二是优化提升农海产品加工产业。加快农海产品加工基地建设，进一步完善加工、保鲜、储存、流通等产业链条，引进先进技术、工艺、设备和管理，推进深加工，提高附加值；重点扶持湛江农垦、恒兴、国联等骨干企业提升产业化、集约化水平，增强龙头带动作用，加快形成农海产品加工产业带。三是做优做强以电饭锅为主导产品的家电产业。重点培育廉江、坡头和吴川三个电饭锅生产基地，调整产品结构，提升技术层次，打造知名品牌，保持电饭锅生产能力和市场占有份额居全国之首。四是加快发展造纸产业。以70万吨木浆项目建设为契机，以湛江晨鸣、冠豪等20多家规模以上造纸及纸制品企业为骨干，推动产业协作配套，加快规模扩张，打造林浆纸一体化生产基地。五是努力振兴纺织产业。以湛江纺织企业集团、大中纺织企业等为龙头，加强产业延伸配套，形成上、下游密切关联的纤维、纺纱、织造、印染、服装等垂直型产业链。六是不断壮大制糖产业。积极支持大型糖业集团实行公司加基地经营模式，扩大深加工和综合利用，提高综合效益，大力发展“吨糖田”，保持年产糖量100万吨。推动机电、木制品加工、制药、食品、饮料、羽绒等产业实现集约发展，壮大产业规模。

　　推进企业自主创新提升工业发展水平。坚持走以市场为导向、以企业为主体、以人才为根本、以产业技术为重点的自主创新路子，突出抓好重点区域、重点产业、重点企业和重点产品的技术改造与创新，逐步形成区域龙头示范、重点产业支撑、骨干企业引领、名牌产品带动的自主创新格局。支持一批行业龙头企业建立技术中心，加大研发投入，提高自主研发能力。争取年内组建市级以上企业工程中心3家、技术开发机构5家，专利申请量和授权量分别增长10%以上。积极筹建广东省海洋开发研究院。深入实施品牌战略，鼓励更多企业主动参与国际国内标准化活动，争创更多名牌产品、著名商标。推进一批电子信息、生物医药、新能源、新材料等高新技术项目建设，促使更多工业企业转向生产高新技术产品，全市高新技术产业产值增长20%。广泛运用高新技术和先进适用技术改造传统产业，重点支持20个促进产业升级、实现技术跨越的技改项目。

　　推进县域工业化培育经济强县。实施工业强县战略，按照县域经济特色化、特色经济产业化、产业发展规模化的思路，以项目为龙头，园区为载体，优势骨干企业为支撑，提升县域工业化水平。充分发挥各县（市）的优势，加快建立农产品加工体系和优势资源型工业体系，发展壮大特色产业，培育优势企业和名牌产品。支持重点项目在县域布局，选择一批有资源优势和市场前景，对财政增长、农民增收效益较好的工业项目进行重点扶持。对具备一定规模、经营业绩良好的优势骨干企业，创造条件促进其加快发展，成为带动县域工业的龙头。每个县（市）着力培育1至2个优势产业集群，3至5个拥有较高市场份额的拳头产品，5至10家纳税超千万元的骨干企业。支持有关县（市）申报建立省级产业集群示范区、省级产业转移工业园和省级特色产业基地，推进布吉（吴川）、金湾（官渡）和顺德（廉江）等产业转移园区建设，全面展开招商引资，力争年内一批产业转移项目落户园区或建成投产。

　　二、加快发展现代农业，建设社会主义新农村

　　着力构筑现代农业产业体系。坚持以市场为导向，调整优化农业经济结构，大力发展优质、高产、高效、生态、安全农业，加快推进优势农产品的区域化布局、规模化生产和产业化经营。在促进粮食稳定发展的基础上，重点抓好糖蔗、红橙、菠萝、北运菜、花卉、南药、甜玉米等10大优势农产品产业带建设，发展壮大对虾、珍珠、果菜、畜牧、热作、林木等特色产业链，建设完善生产加工、储藏运输和市场营销体系。全面实施《农产品质量安全法》，推动农业标准化生产，建设农业标准化示范区和无公害产品、绿色食品、有机食品以及地理标志保护产品基地，提高农产品质量安全水平。加强对台农业招商，加快海峡两岸农业合作试验区建设。推进渔业生产现代化，建设国家海洋产业基地调顺岛示范区、粤西星火技术水产带和国家级海产品质量监督检验中心，建立一批技术含量高、带动能力强的海水养殖和渔业深加工示范基地。推广珍珠科技养殖，提高精深加工水平，振兴南珠产业。大力发展农业龙头企业，完善龙头企业和农户的利益共享机制，建立双方互惠稳定的互动型发展关系。认真贯彻落实《农民专业合作社法》和《广东省农村集体经济组织管理规定》，建立和完善农村集体经济组织，加快发展多种形式的农民专业合作社。

　　着力提高农业综合生产能力。进一步强化现代农业的科技支撑，坚持用现代物质条件装备农业，用现代科学技术改造农业，不断提高农业水利化、机械化和信息化水平，提高土地产出率、资源利用率和劳动生产率。实施“沃土工程”，认真做好675万亩基本农田保护工作。推进建设雷州青年运河灌区节水改造、鉴江供水枢纽、雷州半岛扩大治旱、节水灌溉和中低产田改造等重点工程，年内完成13宗城乡水利减灾防灾工程。加快22宗水库除险加固、23宗灌区改造和80宗引水工程建设，解决53万人饮水安全问题。加强交通枢纽和农村运输节点的连接，提高农村公路网的通达深度和服务水平，年内建设4个公路主枢纽、47个县镇客运场站及500个行政村公交站点；建成镇通村委会公路路面硬化900公里，新建改建交通桥梁30座，通车行政村达到1100个。强化农业信息技术服务，实现网络进村、信息入户。推进硇洲、乌石国家级中心渔港和人工渔礁建设。

　　着力推进社会主义新农村建设。贯彻落实中央关于社会主义新农村建设的总体要求，立足当前，着眼长远，尊重农民意愿，注重实际效果，以“六个一”为载体，即制定一个合理规划，发展一个特色产品，推进一批改造工程，建设一个公共村场，营造一个洁净环境，种上一片成荫绿树，以解决农村“一保五难”为突破口，把新农村建设不断引向深入。坚持以规划为龙头，因地制宜搞好乡村规划建设，优化村庄布局，集约使用土地，突出地域特色。大力推广各地、各种类型生态文明村建设经验，培育和推出一批新农村建设典型和样板，发挥示范引导效应，以点带面。引导广大群众当主人、作主体、唱主角，继续推广和完善“企业家回归”、“村企共建”等多种模式，广泛动员民营企业家等社会力量参与新农村建设。在全市开展10个农业生态经济区、10个最整洁乡镇、100个带动农户致富经济组织、100个最美村庄、1000个农业种养能手、1000个最美农家庭院评选活动，推动新农村建设向更广的范围和更高的水平发展。

　　三、提升第三产业发展水平，努力扩大消费拉动

　　推进港口物流中心建设。充分发挥港口优势，加快建设货运中心、配送中心、物流信息中心和商品交易中心，努力拓展综合批发、期货交割、保税中转等物流业态，着力打造区域性物流中心。完善港口货物调运、加工、仓储、分拨、转口贸易等配套设施，拓展港口运输业，开通湛江至中西部各主要城市集装箱海陆联运快线，扩大集装箱运输和中转业务。按照“前港后库”、“前港后厂”的模式，推进港口物流园区建设，形成油品、矿石、化肥、粮食、钢材等区域物流聚集中心。发展一批以港口贸易为龙头，实行外贸、内贸、生产、科研一体化，经营规模合理、技术装备水平较高的物流企业集团。进一步完善港口服务功能，加快物流信息体系建设，积极推进跨区域通关业务，提高港口物流效率和发展水平。力争全市港口货物吞吐量达到9000万吨。

　　推进区域性商贸流通中心建设。实施《湛江市商业网点发展规划》，推动一批购物、餐饮、娱乐、旅业大型项目建设，改造提升一批重点商业圈，着力培育城市中央商务区。着重改造提升零售、餐饮、农贸市场等传统服务业，形成便民利民的社区服务网点；大力发展信息、金融、保险、会展等现代服务业；促进发展文化、中介、社区等新兴服务业；积极发展与先进制造业紧密融合的生产性服务业。以市场化运作为手段，扩大连锁经营、电子商务和第三方物流，培植壮大一批商贸流通骨干企业。加快发展农副产品、对虾、粮食、农资、花卉等一批集聚辐射能力强的区域专业市场，规划建设湛江水产城，继续办好对虾节。有序发展房地产业，带动装饰装修、物业管理等相关行业发展。年内服务业增加值增长13%。

　　推进区域性商旅中心建设。以游促商，商旅结合，积极引入外资和社会资本开发经营旅游项目，加快旅游基础设施和旅游资源开发建设。突出蓝色滨海休闲、绿色自然生态、红土民俗文化特色，着力整合全市旅游资源，打响“彩色湛江”旅游品牌。大力开发适应市场需求的各类旅游产品，举办形式多样的专题旅游节庆活动，有针对性地开展旅游市场推介和促销。积极支持湖光岩风景区建设，推进中国雷琼世界地质公园旅游区域的协作，扩大世界地质公园品牌效应；加强与泛珠三角等区域旅游合作，以“互为市场、互送客源、互利共赢”为原则，拓展旅游客源市场，扩大旅游业发展空间。

　　四、强化基础设施和城市规划建设，构筑跨越发展的坚实平台

　　加强城市规划工作。充分发挥城市规划对城市发展的指导、统筹和综合调控作用，强化空间管治，形成较为科学的功能分区、畅通的交通网络、完善的绿地系统，为城市的持续健康发展奠定良好基础。完成东海岛和坡头海东新区控制性详细规划编制，修编麻章区、疏港公路等主干道两侧及城中村控制性详细规划，完成教育、城市交通、电力、市容环境卫生和主城区地下空间开发利用等专项规划。启动《湛江港湾区域建设用海总体规划》编制，指导和规范科学用海。实施《城市规划管理办法》和《城市规划管理技术标准与准则》，规范规划修编和实施程序，切实提高规划管理水平。推进城市规划信息化建设，建立市区数字地形图。实施城市规划效能监察活动，加强对规划工作的公开监督，坚决查处违法建设行为，确保各项规划有效实施。

　　构建以港口为中心的大交通体系。抓住湛江港被国家确定为西南沿海主要港口和我市被列入全省4个综合性运输枢纽建设重点城市之一的契机，大力加强交通集疏运的衔接配套，构建层次分明、功能完善的综合运输枢纽，形成大交通格局。立足建设南方大港、冲刺亿吨目标，加快建设湛江港30万吨级航道、宝满集装箱码头等8大港口工程，实施调顺、霞山港区技术改造。积极推进流沙、海安、乌石等县域港口建设，促进环雷州半岛港口群协调发展。促进建设湛徐高速公路和海湾大桥西连接线湛江至雷州一级公路，扩建东海大堤，启动建设疏港公路二期工程和东海岛疏港公路，实施325国道绕市区段改线和广湛高速公路官渡入口平交改立交工程，加快筹建坡头至官渡高速公路出入口快线及南三大桥。全力支持黎湛铁路河唇至湛江段增建二线和粤西沿海铁路湛江段建设，加快洛湛铁路茂湛段、合河铁路前期工作，力争将东海岛铁路作为茂湛铁路延伸段列入省重点项目尽快动工。

　　推进城市基础设施建设。进一步完善市政道路网络，续建百蓬路、椹川东二路、体育南路、东盛路和南方路网工程，改造建设奋勇大道、北站路、站前路和北进出口道路。优先发展公共交通，加快市区客运枢纽站场和公交候车亭等设施建设。推进霞山汉口路片旧城和城中村、城郊村改造，加快改造市区小街小巷和城市主要进出口道路。以建设生态园林城市为目标，进一步优化城市人居环境。建成南国热带花园、中澳友谊花园和绿塘河公园，继续改造三岭山森林公园，启动建设滨湖公园和海湾大桥桥东公园，推进一批城市道路绿化改造和小绿地、小游园建设，年内市区新增绿地100万平方米以上。启动市区供水备用水源建设。

　　加快城镇化进程。坚持“规划共绘、设施共建、产业共树、资源共享、生态共保、优势共创”，充分发挥市、区、镇共同推进城镇化的积极性。全面落实省、市加快中心镇建设的各项政策措施，重点建设条件好、发展潜力大的县（市）城区、中心镇和专业镇，形成产业发展、人口聚集、市场活跃的经济社会要素集聚区，增强对广大农村的辐射带动作用；完善建制镇总体规划，科学定位，进一步明确发展重点、步骤、措施，以基础设施和特色产业建设为突破口加快发展，逐步构建以市区为龙头、以县（市）城区和中心镇为主轴、以建制镇和农村中心村为基础，布局合理、组合有序、优势互补、整体协调、持续发展的城镇体系。

　　提高城市管理水平。积极探索城市网络化、精细化管理模式，完善“二级政府、三级管理、四级网络”的城市管理体制，按照“重心下移”和“权责一致”的原则，构建“统一领导、分级负责、条块结合、依法管理、社会监督”的城市管理格局。进一步加强城市管理基础建设，广泛创建“六好”平安和谐社区。推进园林、环卫管理市场化、社会化改革，落实市容环境属地管理责任制，强化城市公用设施维护和管理，坚决拆除违法违章建筑。优化交通组织，均衡交通流量，规范交通秩序，提高道路通行效率。进一步提高城市管理综合执法效能，积极探索建立公安协同城市综合执法机制。完善城市管理热线投诉系统。

　　五、深入推进各项改革，加快经济市场化进程

　　以国有资产保值增值为重点推进国有企业改革。坚持有所为有所不为，加快建立国有资本有进有退、合理流动机制。积极推进资产优化重组，加快产权多元化进程，盘活国有企业存量资产，化解历史遗留问题和债务负担；支持市属国有优势企业引入高品位战略合作伙伴，力促国有劣势企业退出市场。建立国有资本经营预算制度，完善国有资产授权经营、国有企业年度经营和任期经营业绩考核责任制，规范国有企业负责人薪酬，建立有效的激励约束机制。积极探索市场化选聘国有企业经营管理者的方式与途径。

　　以完善公共财政体系为重点推进财税体制改革。进一步规范财政国库管理，集中支付逐步涵盖预算外资金，实施市直财务核算集中监管。调整财政支出结构，更多地向公共服务、社会保障倾斜。强化政府采购管理和监督，扩大政府采购规模和范围，完善财政支持的工程项目纳入政府采购实施办法。积极构建财政支出绩效评价体系，逐步推进绩效预算，对使用市级财政资金100万元以上的项目预算单位实行自我绩效评价和项目绩效预算申报，切实提高财政资金使用效益。努力构建行政事业性资产管理新体系，加强对政府垄断性经营服务收入和社会公共资源经营收益的管理。大力推进财源建设，完善财政激励制度，进一步激发各地发展活力。坚持依法治税，强化收入征管，促进财政收入稳定增长。

　　以优化结构提高效益为重点推进投资体制改革。完善投资管理制度，实行更加便捷有效的项目备案制、核准制和审批制。突出企业的投资主体地位，营造各类投资者公平有序的竞争环境，打通政府资金需求与社会资金供给之间的通道。规范政府投资行为，建立投资决策责任制，实行投资项目公示和评估制度。探索政府资本市场化运作新模式，以市场机制和手段对公共资源进行集聚、整合和经营。推行非经营性政府投资项目代建制。

　　以改善环境优化服务为重点加快非公有制经济发展。全面贯彻落实《国务院关于鼓励支持和引导个体私营等非公有制经济发展的若干意见》，积极为非公经济加快发展营造良好的法制、政策和市场环境。通过政策支持做强一批骨干民营企业；通过放宽准入发展一批新兴民营企业；通过招商引资引进一批外地民营企业。进一步完善中小企业服务体系，设立民营企业信用担保基金，鼓励信用担保机构为民营企业开展贷款担保业务。

　　以建设新型银政企关系为重点推进金融改革和发展。完善金融服务功能，促进金融与经济发展的良性互动，构建良好的金融生态环境，努力创建金融生态示范市。坚持金融机构联席会议制度，搭建银政企合作平台。推动金融服务和金融工具创新，促进发展信贷、股票、债券、信托、票据等多种融资形式，积极发展资本、保险和期货市场。大力培育上市公司后备资源，支持符合条件的企业加快上市融资。全力支持市商业银行加快发展，继续推进农村信用社管理体制和产权改革。

　　六、努力扩大对外开放，增强经济发展活力

　　积极有效吸收外资。强化吸收外资导向，提高吸收外资质量，大力引进对产业升级具有重大带动作用的大项目、大企业；结合我市资源优势和产业优势，积极吸引外资投向临港重化、现代农业和农海产品深加工、先进制造业、高新技术产业及现代服务业。创新招商引资形式、方法和手段，加快建立以企业为主体的招商机制。精心策划、包装、推介招商项目，落实用地、用水、用电、厂房建设等招商条件，增强招商项目的可行性和可操作性，提高招商引资实效。积极做好招商引资项目的跟踪服务工作，提高实际履约率。严格落实招商引资目标责任制和鼓励政策，充分调动各级招商引资的积极性，县（市、区）实际吸收外资实现较大幅度增长。

　　促进外贸持续发展。大力实施“科技兴贸”战略，加快转变对外贸易增长方式，优化出口商品结构，努力扩大技术含量高、附加值高的产品出口，提高传统出口商品质量，推动加工贸易转型升级，大力发展服务贸易。积极培育多元化外贸经营主体，支持更多企业取得进出口经营权。完善出口促进和鼓励政策，着力培植具有一定规模、市场竞争力强的出口龙头企业。进一步完善外贸促进服务体系，加强与海关、检验检疫、海事、边检等部门的沟通和协作，推进电子口岸二期工程建设，进一步完善口岸查验配套设施，构建便捷高效的口岸“绿色通道”，提高通关效率。大力支持有条件的企业到国外投资，参与国际合作与竞争。积极应对国外技术壁垒和绿色壁垒，提高抵御市场风险的能力。

　　加强区域经济合作。主动参与中国—东盟自由贸易区、港澳地区、珠三角、大西南和环北部湾地区的经济技术合作，善抓发展商机，扩大发展空间。充分利用我市成为大西南出海主通道的有利条件，推动优势企业与中西部企业的战略合作，促进资源、市场与资金、技术、品牌共享。积极促进与澳大利亚凯恩斯市、俄罗斯谢尔普霍夫市等国内外友好城市之间的交流与协作，提升合作水平。办好2007湛江工业博览会。

　　七、加强资源节约和环境保护，建设资源节约型和环境友好型社会

　　推进资源节约和综合利用。坚持开发与节约并重，节约优先，推进节能节水节地节材工作，重点抓好石化、电力、建材、纺织等重点行业和100家重点耗能企业的节能降耗和能源计量管理，建立投资项目节能评估和审查制度，依法淘汰落后工艺和产品。年内单位工业增加值能耗降低2.75%以上，工业固体废物综合利用率达88%以上。推进工业生产中水回用工程建设，完成制糖业节水技术改造，推广农业节水灌溉设施技术，鼓励建筑业扩大使用再生材料、替代材料、新型材料。大力推进节约集约用地，认真执行工业项目建设用地控制指标；加强土地收购储备，盘活闲置土地资源。加快实施水、电、油、气等资源性产品价格改革，促进资源节约。全面推进节约型企业、节约型机关、节约型单位创建活动。

　　大力发展循环经济。按照“减量化、再利用、资源化”原则，以企业为主体，政府推动、市场引导、公众参与相结合，实现企业、园区、社会三个层次循环经济的互动发展。鼓励循环经济技术的研发和推广应用，实施循环经济示范工程，开展以电力、石化、制糖企业为重点的循环经济项目试点工作。建立再生资源回收利用市场。

　　加强生态环境保护和建设。全面实施省、市环境保护规划。严格执行国家《环境影响评价法》，认真开展对各类规划和建设项目的环境影响评价，提高环保准入水平。推进创建国家卫生城市、国家环保模范城市工作。深入实施碧水、蓝天、宁静、洁净、生态和治污保洁工程。加强九洲江、鉴江、南渡河等重点流域和天然水体、农村水网以及地下水资源的保护利用。强化海洋环境保护，鼓励发展生态健康养殖业，建立雷州湾中华白海豚保护区。落实减少污染物排放总量控制责任，确保完成省下达的控制任务，化学需氧量和二氧化硫排放量分别降低5.4%和5%。继续实施绿塘河、文保河、南柳河和赤坎江整治二期等排水截污工程；完成霞山污水处理厂、赤坎污水处理厂二期工程，动工建设麻章污水处理厂，筹建坡头、东海岛污水处理厂和市区生活垃圾场污水处理扩容等工程。大力推广使用清洁能源，加快发展节能环保型公交，年内投入100辆液化天然气公交车运营。鼓励利用太阳能和使用沼气。积极推行清洁生产，争取年内5家企业通过省审核。强化餐饮业油烟和机动车尾气污染治理，完成湛江电厂脱硫工程，逐步搬迁城市建成区内污染企业，进一步优化城市环境质量。开展全国绿化模范城市创建活动，推进国家级生态示范区、林业生态县、省级生态示范村镇和绿色社区等创建活动，新增造林面积24万亩。

　　八、着力加强社会建设，促进各项事业协调发展

　　优先发展教育事业。坚持教育发展优先，完善义务教育投入保障机制，推进义务教育规范化学校建设。实施“扩容促优”工程，积极发展优质教育资源，推进示范性高中建设。建立城乡支教制度，提高农村师资素质。深入实施市区学校和农村义务教育学校生活设施改造，年底前全部消除农村中小学C、D级危房。在去年农村义务教育免收杂费的基础上，今年秋季免收农村义务教育书本费，在农村实现完全意义上的义务教育。促进发展职业技术教育，建设一批重点职业院校、重点专业和实训中心，加快培养适应经济社会发展需要的技能型人才和实用人才。大力支持在湛高校扩大办学规模，积极推动广东医学院坡头新校区筹建工作。

　　加快建设文化大市。努力培育地方特色文化品牌，加强历史文化遗产和民间艺术传承，妥善保护历史文化名城、街区、乡镇、重要历史建筑和文物。推动社区、企业、校园等文化建设，因地制宜深入开展农村特色文化村、文化室、文化节活动，丰富城乡群众精神文化生活。建成市广播电视中心，筹建市群众文化艺术中心，实施市区数字电视整体转换。培育壮大经营性文化产业，以新的机制发展一批有潜力的文化项目。完善和落实文化经济政策，发展一批具有较强实力和竞争力的文化企业及文化产业集团，壮大文化市场主体。

　　积极发展公共卫生和体育事业。加强公共卫生和医疗服务体系建设，推广建立平价医院、平价病房、平价药店，为低收入群众提供廉价实惠的医疗服务，推行药品网上竞价采购制度，全面调整和规范医疗服务价格，努力解决群众看病难、看病贵问题。建立健全人畜共患重大动物疾病防控机制。推进城市社区和农村卫生基础设施及城乡卫生服务体系建设，继续改造乡镇薄弱卫生院，实施村卫生站规范化建设，广泛开展卫生村、卫生街区和初级卫生保健达标创建活动。完善农村合作医疗制度，适当提高农村合作医疗补助标准，年内实现农村人口参合率70%以上。加快发展中医药事业。坚持群众性体育和竞技体育全面发展，积极备战国内外重要体育赛事，组团参加第六届全国城市运动会。

　　加强人口和计划生育工作。全面落实人口与计划生育政策，严格控制人口增长，稳定低生育水平，提高出生人口素质。坚持人口与计划生育目标管理和层级动态管理责任制，严格执行“一票否决权”制度。完善人口与计划生育技术队伍和服务网络。全面开展乡镇（街道）无政策外多孩出生、村（社区）无政策外出生活动。进一步加强流动人口计划生育管理和服务，广泛开展“关爱女孩行动”，综合治理出生人口性别比失衡问题。

　　统筹发展各项事业。积极推进全民国防教育，做好国防动员、民兵预备役和人民防空工作。深入开展拥军优属活动，做好优抚安置工作，巩固和发展军政军民团结。搞好第二次全国农业普查。抓好民族宗教工作，全面落实侨务政策，依法保护归侨、侨眷合法权益。加快档案信息化建设。完善社会专项事务管理，推进殡葬改革，发展慈善事业，实施地名公共服务工程。扎实做好外事、对台、气象、测绘、防灾减灾以及妇女儿童、残疾人、老龄、修志等工作，进一步促进社会事业协调发展。

　　九、坚持以人为本，构建和谐湛江

　　努力提高市民素质。广泛开展以“八荣八耻”为主要内容的社会主义荣辱观和“爱国、守法、诚信、知礼”的现代公民道德教育活动，大力弘扬社会主义道德风尚，形成团结互助、扶贫济困、礼让宽容的社会风气。以政务诚信、商务诚信、社会诚信为重点，推进社会信用体系建设，营造全社会诚实守信、诚信友爱、祥和文明的氛围。深入开展各类群众性精神文明创建活动。实施“五五”普法规划，营造良好的法治环境。

　　千方百计扩大就业。坚持把发展经济作为解决就业问题的根本途径，实现经济发展和扩大就业良性互动。大力发展劳动密集型产业、服务业、中小企业和民营经济，不断扩大就业容量；加快发展工业园区和城市新区，使其成为吸纳就业的新增长点。强化政府促进就业的公共服务职责，认真落实小额贷款、税费减免及社会保险补贴等多项措施，扶持劳动者采取灵活多样的形式就业。积极扩大职业与技能培训，提高劳动者的就业、转业和创业能力。大力开展充分就业社区活动，力争60%以上社区实现充分就业。完善就业援助制度，实现有就业愿望的新增城镇“零就业家庭”每户至少1人就业。全市新增就业岗位4万个；下岗失业人员再就业1.2万人，其中“4050”人员实现再就业4000人。努力促进农村富余劳动力转移就业，继续推进沿海渔民转产转业培训工作，年内组织农村青年和退役士兵免费技能培训4万人，转移农村劳动力就业15万人。安置残疾人3000名，残疾人就业率达85%以上。

　　加快完善社会保障体系。落实社会保险扩面征缴工作责任制，进一步扩大社会保险覆盖面。完善社会保险市级统筹，推进地税全责征缴。切实加强社会保险基金的监管，健全征缴管理和支出稽核制度，确保基金收支平衡、安全完整、保值增值。进一步完善城乡最低生活保障制度，筑牢企业退休人员基本养老金、企业职工最低工资、低收入困难家庭补助、失业人员失业保险金和城乡居民最低生活标准等五道保障防线。年内全市企业养老保险增加6万人、失业保险增加2万人、工伤保险增加3.5万人、医疗保险增加2.6万人。进一步完善住房保障制度，加快发展经济适用房和廉租房，着力解决中低收入家庭的住房困难。抓好整村推进扶贫工作，继续实施农村危房改造，年内解决1500户贫困群众住房困难，改造水库移民危房5000户。新建村级敬老院120间。

　　切实维护社会安全稳定。建立全市公共应急处置机构和雷州半岛气象、水文、地质等灾害监测预警系统，提高处置突发事件及应对重大自然灾害能力。落实安全生产责任制，强化重点行业、重点部位和重点设备专项整治和重大安全隐患整改，有效遏制和防范重特大安全事故发生。继续实施食品药品放心工程，健全和规范食品市场准入制度，完善农产品检测网络，加强食品污染物、有害物和药品监测，确保食品和用药安全。规范市场经济秩序，制止不正当竞争，坚决打击制售假冒伪劣商品、非法传销以及交通、农产品流通等领域和地区封锁的非法垄断行为。加强价格监控，防止价格异动。完善社会利益协调和社会纠纷调处机制，形成人民调解、行政调解、司法调解相衔接的大调解格局；加强司法工作，维护司法公正；进一步落实信访工作责任制，依法规范信访秩序，坚持领导下访、约访制度，努力把各类矛盾解决在基层和萌芽状态。健全完善村民自治机制，促进基层民主法制建设。推进城乡治安防控体系建设，健全城市社区、乡村警务网络，促进治安管理和防范社会化；依法严厉打击“两抢一盗”等严重违法犯罪和黑恶势力，保持打击走私高压态势，坚决扫除黄赌毒等社会丑恶现象；切实加强对敌斗争，完善反恐工作协调机制，维护国家安全和社会稳定。

　　十、加强政府行政能力建设，努力提高政府工作水平

　　进一步推进依法行政。全面实施《行政许可法》和国务院《全面推进依法行政实施纲要》，理顺行政执法体制，完善行政执法程序，规范行政执法行为，界定行政执法责任，保证各级行政机关及其工作人员严格按照法定权限和程序行使职权、履行职责。各级干部要带头学法、守法、用法，善于运用法律思维方式处理问题，解决矛盾，推动工作。贯彻实施《公务员法》，努力建设一支想干事、敢干事、会干事、干成事的公务员队伍。严格规范性文件的制定程序和备案审查，搞好行政复议，依法开展行政应诉。自觉接受人大及其常委会、政协、各民主党派和人民团体、人民群众和新闻舆论的监督，积极办好人大代表议案、建议和政协委员提案。

　　进一步强化行政责任。建立健全主体明确、层级清晰、操作性强的岗位责任制，制定职位说明书，明确工作责任和工作规程；完善目标责任制和主办负责制，构筑层级之间、岗位之间的责任链条。加快建立以行政领导为重点的行政问责制，大力推进考核监督的制度化、规范化；把行政问责与行政监督、审计监督结合起来，制定和完善质询、罢免、引咎辞职、责令辞职等相关配套制度。实施行政审批电子监察。对责任不到位、目标不完成、承诺不兑现，行政不作为、乱作为，影响行政效能，贻误行政工作等行为，要严肃追究领导责任、单位责任和当事人责任。

　　进一步转变政府职能。深化行政管理体制改革，推进政府管理创新，建设服务型政府。政府管理要把主要精力放在为各类市场主体服务和创造良好发展环境上，在更好地履行经济调节和市场监管职能的同时，更加注重履行社会管理和公共服务职能。进一步推进政企分开、政资分开、政事分开、政府与市场中介组织分开，凡是政府应承担的责任，做到责无旁贷；凡是属于企业和社会自主决策的，政府决不承揽包办；凡是能用市场办法解决的，就不用行政手段。大力推进决策科学化、民主化，完善重大决策调研、专家咨询论证、社会公示听证制度。深化行政审批制度改革，进一步简化和规范审批程序，推进网上审批；加强市、县两级行政服务中心建设。大力推进政务和信息公开，完善政府公报、政府网站、新闻发言人等公开渠道，全面改造提升市区政务公示设施，加快电子政务建设，提高政府工作的透明度。

　　进一步提高决策执行力。加快建立高效快捷、迅速果断的执行机制，做到有事则议，议则早定，定了快办，办则办好，确保各项决策及时全面贯彻执行。大力弘扬求真务实、苦干实干的精神和作风，对上级作出的决策、提出的工作目标任务，各地各部门要拿出针对性、指导性、操作性强的工作方案和具体落实措施，精心组织实施，务求一抓到底，抓出成效。各级政府和全体政府工作人员要牢固树立以人为本、执政为民的理念，努力实现好、维护好、发展好广大人民群众的根本利益，真心实意为人民群众办实事、做好事、解难事，全心全意为人民服务，以锐意进取、开拓创新的精神风貌取信于民，以勤勉尽责、真抓实干的工作作风取信于民，以坐言起行、励精图治的实际行动和工作成效取信于民。

　　进一步加强廉政建设。坚持标本兼治、综合治理、惩防并举、注重预防的方针，建立健全与新形势、新环境、新任务相适应的教育、制度、监督并重的惩治和预防腐败体系。进一步完善工程招投标、土地出让、产权交易、政府采购以及领导干部经济责任审计等制度，加强对重要部门、重要岗位和关键环节权力行使的监督。推进政风行风建设，坚决纠正各种损害群众利益的不正之风。认真贯彻执行国务院《党政机关国内公务接待管理规定》，规范公务接待行为。加大治理商业贿赂工作力度，严肃查处以权谋私等违纪违法行为。

　　各位代表，我们肩负着党的重托和人民的期望，踏上了湛江跨越发展的新征程。湛江人民正迸发炽热的创业激情，湛江大地正焕发蓬勃的发展生机，未来的湛江充满机遇，充满希望。让我们紧密团结在以胡锦涛同志为总书记的党中央周围，在省委、省政府和市委的正确领导下，万众一心，拼搏进取，攻坚克难，创业图强，勇当广东东西两翼跨越发展的排头兵，为在广东区域协调发展大格局中率先崛起，与全省同步率先基本实现社会主义现代化而努力奋斗！
